# Growth patterns of *Pseudomonas aeruginosa* in milk fortified with chitosan and selenium nanoparticles during refrigerated storage

**DOI:** 10.1007/s11274-023-03757-3

**Published:** 2023-09-21

**Authors:** Rehab M. Atia, Hamdi A. Mohamed, Nahla A. AboELRoos, Dina A. B. Awad

**Affiliations:** 1 Shebin El Koom branch, Animal Health Research Institute, Giza, Egypt; 2 Food Hygiene and Control Department, Faculty of Veterinary Medicine, Benha University, Moshtohor, Qalyubia, 13736 Egypt

**Keywords:** Psychrotrophic bacteria, Dairy industry, Food safety, Nano-chitosan, Nano-selenium

## Abstract

*Pseudomonas* spp are considered a common milk-associated psychotropic bacteria, leading to milk deterioration during storage; therefore, our study aimed to study the distribution of *Pseudomonas aeruginosa* in raw milk and its associated products then studying the growth behavior of *P. aeruginosa* in milk after employing chitosan nanoparticles (CsNPs 50, 25, and 15 mg/100ml) and selenium nanoparticles (SeNPs 0.5, 0.3 and 0.1 mg/100ml) as a trial to control the bacterial growth in milk during five days of cooling storage. Our study relies on the ion gelation method and green synthesis for the conversion of chitosan and selenium to nanosized particles respectively, we subsequently confirmed their shape using SEM and TEM. We employing *Pseudomonas* selective agar medium for monitoring the bacterial growth along the cooling storage. Our findings reported that high prevalence of *Pseudomonas spp* count in raw milk and kareish cheese and high incidence percent of *P. aeruginosa* in ice cream and yogurt respectively. Both synthesized nanoparticles exhibited antibacterial activity in a dose-dependent manner. Moreover, CsNPs50 could inhibit the *P. aeruginosa* survival growth to a mean average of 2.62 ± 1.18 log_10_cfu/ml in the fifth day of milk cooling storage; also, it was noted that the hexagonal particles SeNPs0.5 could inhibit 2.49 ± 11 log_10_cfu/ml in comparison to the control *P. aeruginosa* milk group exhibited growth survival rate 7.24 ± 2.57 log_10_cfu/ml under the same conditions. In conclusion, we suggest employing chitosan and selenium nanoparticles to improve milk safety and recommend future studies for the fate of nanoparticles in milk.

## Introduction

Globally, food safety is a critical problem impacting food technology and public health. Among the potential issues are food contamination and foodborne diseases (Belli et al. 2013). Milk and dairy products are considered a high-risk category for potential microbial contamination, resulting from processing techniques, livestock and the surrounding environment, all of which can contribute to the contamination (Washabaugh et al. 2019).

Milk deterioration might occur at any step from the farm to the consumer, even after being thermally heat treated. The milk storage in refrigerators supports the growth of psychrotrophic bacteria, which can thrive below refrigerated temperatures of 4–7 °C and form heat-resistant bacteria. Psychrotrophic bacteria can form hydrolytic enzymes such as lipases, proteases, and phospholipases. These enzymes resist high temperatures and cause milk spoilage by changing milk’s physicochemical and sensory properties, leading to economic loss. The hydrolysis of casein by protease enzymes leads to milk coagulation and undesirable changes in the flavor of liquid milk and dairy products, such as bitterness, metallic taste, and rancidity (Tchorbanov et al. 2011).

*Pseudomonas spp* is a psychrophilic bacterium that promotes the deterioration of most dairy products and other food items when kept at 4 °C or refrigerated during transportation (Carminati et al. 2019; Wang et al. 2018). It can produce proteases and lipases that can withstand severe thermal treatment but degrade milk proteins and fats, resulting in bitterness, rancidity, and gelation, which has a significant negative impact on the quality of milk and dairy products (Jaspe et al. 2000; Yao et al. 2012).

The opportunistic pathogen *Pseudomonas aeruginosa* causes deadly diseases such as sepsis and pneumonia. It frequently shows different resistance mechanisms to multiple antibiotics (Abd El-Baky et al. 2020). Different *pseudomonas Spp* as *ps. aeuroginosa, ps. Fluorescence, ps. Putida, ps.diminuta*, and *ps. Fragi* could be isolated from different milk products, including raw milk, kareish cheese, yogurt, and ice cream (Atia et al. 2022). As a result, it is crucial to consider novel ways to produce safe milk products, such as the application of nanotechnology, which is an attractive future candidate for chemical preservation (Wang et al. 2017).

A unique antimicrobial strategy uses natural-derived components to inhibit the growth of foodborne illness and spoilage bacteria and enhance the food organoleptic properties (Bajpai et al. 2013; Xu et al. 2017). The focus on improving the safety of food production and processing chains has increased during the past 20 years (Satterthwaite et al. 2010). Nanotechnology is a cutting-edge multidisciplinary field showing recent rapid development in several applications. Researchers and the industrial sector have identified potential uses of this technology in nutraceuticals and functional foods to enhance human health (Pradhan et al. 2015).

Chitosan, which is generated from the exoskeletons of crustaceans, invertebrates, and fungi, is the second most prevalent biopolymer in the world. Chitosan is the deacetylated product of chitin and is made up of haphazardly distributed molecules of N-acetyl-d-glucosamine and -(1-4)-linked d-glucosamine in the linear cationic polysaccharide chain (Ruocco et al. 2016). It serves a variety of biological uses and is biocompatible, safe, and degradable; therefore, it was approved as generally recognized as safe (GRAS) by the US Food and Drug Administration (US FDA) (Garg et al. 2019). According to Sayari et al. (2016), chitosan has several antibacterial activities and can kill most microorganisms, such as Gram-negative and Gram-positive bacteria, fungi, and yeast.

Chitosan’s physical characteristics can be enhanced, and its industrial applications expanded by adopting an appropriate nanoparticle synthesis process. Chitosan nanoparticles (CsNPs) have better physicochemical properties than chitosan (Chandrasekaran et al. 2020).

Selenium (Se) is a crucial trace element that should be included in the human diet for well human health and is also considered an active component of various body enzymes (Zhang et al. 2018).

Selenium nanoparticles have received more interest recently due to their great bioavailability and low toxicity (El-Sayed et al. 2020). Its unique physical and chemical properties make it appealing for various technological applications (Geoffrion et al. 2020). Selenium nanoparticles could trigger various application in food and agriculture as antimicrobial and therapeutic theraby and functional packaging (Garza–García et al. 2022).

Therefore, the main objective of the current study was to investigate the prevalence of *Pseudomonas* aeruginosa in raw milk, and its associated dairy products ( kareish cheese, yogurt, and ice cream) then examine the antibacterial functionality of different chitosan (CsNPs) and selenium (SeNPs) nanoparticle different concentrations against psychrotrophic *Pseudomonas aeruginosa* bacteria in milk as a trial to control its growth by natural bio-preservatives.

## Materials and methods

### Materials

Bacterial reference strain *Pseudomonas aeruginosa* ATCC 27,853 was obtained from Animal Health Research Institute, Doki, Egypt. The growth medium used was tryptic soya broth and *Pseudomonas* selective agar medium supplemented by glycerol obtained from (HiMedia Chemicals Pvt. Ltd, Mumbai, India). The reagents and materials used were as follows: chitosan (with purity > 90%), sodium selenite hydrate 99% (Na_2_SeO_3_), and glacial acetic acid was purchased from Sigma-Aldrich ( St. Louis, MO, USA). All other chemicals used in this study were of analytical grade.

Our protocol depends on two steps, firstly, survey study on the prevalence of *Pseudomonas spp* in different milk and milk products samples. Secondly, a trial to control *Pseudomonas aeruginosa* in milk using natural preservatives.

### Sample collection and Pseudomonas spp isolation

A total of 100 samples from raw milk, kareish cheese, yogurt, and ice cream (25 sample each) were randomly collected from Monofiya groveronorates, Egypt. The collected samples transferred in ice tank and rapidly transferred to the laboratory. Counting of *Pseudomonas spp* was on *Pseudomonas* selective agar medium supplemented by glycerol and incubated at 37 °C for developing a greenish yellow colony. Based on colony morphology and Gram stain, we select single colony for further identification.

### Identification of * Pseudomonas aeruginosa*

The selected purified colonies subjected to biochemical test (ISO, 2004). The positive *Pseudomonas aeruginosa* subjected to molecular identification using polymerase chain reaction after extraction of DNA ( Wizard Genomic DNA Purification Kit, Promega Corporation, Madison, WI), for identification the virulence genes presence (oprl, txoA and opSl) according to Atia et al. (2022).

### Synthesis of chitosan nanoparticles

As described by (Piras et al. 2015) and (Hosseini et al. 2018), chitosan nanoparticles (CsNPs) were synthesized by the process of ionic gelation. Chitosan was dissolved in aqueous acetic acid with 1% (v/v) at a concentration of 3 mg/ml and kept under continuous stirring overnight to ensure the chitosan was dissolved entirely. The pH was then adjusted to 4.8 ± 0.02 with 1 N NaOH. 25 ml of chitosan solution was mixed constantly while 10 ml of tri poly phosphate (TPP at concentration 1 mg/ml) was added dropwise. The CsNPs suspension was then freeze-dried after centrifuged for 30 min. at 750 rpm.

### Synthesis of selenium nanoparticles

Using a green synthesis technique that depends on reducing sodium selenite to prepare selenium nanoparticles (SeNPs) with regard to some modification according to (Chung et al. 2020; Yuan et al. 2023) who depend on coating selenium nanoparticles by Bovine serum albumin (BSA). 100 ml of vitamin C (Ascorbic acid 0.1 M) was slowly dropwise into the 200 ml Na_2_SeO_3_·5H_2_O (5 mM) contains 10 mg/ml BSA. The reaction was conducted under continuous magnetic stirring and finished after 24 h in darkness at room temperature until SeNPs was prepared. After adding the ascorbic acid, this mixture’s color changed from colorless to reddish–orange after 24 h. The mixture was centrifuged for 15 min. at 8000 rpm, the residue was removed by repeatedly rinsing with deionized water, and the recovered particles were freeze-dried then at using dissolved under sonication (Shahabadi et al. 2021).

### SEM and TEM investigations of the prepared nanoparticles

The surface morphology, size and shape of CsNPs and SeNPs were examined by employing scanning electron microscopy (SEM), and samples of the prepared nanoparticles were coated with gold using a sputter coater (model JEOL-JSM-IT200; with an acceleration voltage of 20 kV”). Before the analysis, the samples were placed in an ultrasound bath for 15 min and 1 or 2 drops were deposited in a 200-mesh copper grid, suspended for 10 min, followed by removing the excess sample. The grid remained in the desiccator for at least 24 h. A transmission electron microscope (TEM) “JEM-2100 Plus, JEOL Ltd., Japan, also investigated the prepared nanoparticle morphology.

### Application of prepared nanoparticles in the artificially contaminated milk

#### Obtaining and preparation of * Pseudomonas aeruginosa*

*Pseudomonas aeuroginosa* (ATCC 27,853) used in this study was obtained from the Media Unit, Food Hygiene Department, Animal Health Research Institute, Dokki, Giza, Egypt. The identified *Pseudomonas aeruginosa* was activated on tryptic soya broth for 3.5% and then adjusted (~ 5 log cfu/ml).

#### Milk inoculation

Raw skimmed buffalo milk was collected from the herd of Menofia University at Egypt Faculty of Agriculture. Raw buffalo milk was subjected to ultraviolet light treatment (wavelength 254 nm) for 20 to 30 min. after being promptly transported to the lab in an icebox at 4 °C to eradicate microflora (Bintsis et al. 2000).

Treated raw buffalo milk was artificially inoculated with *Pseudomonas aeruginosa* (~ 5 log cfu/ml). Then, milk was thoroughly mixed until even the distribution of microbes before being left for 30 min to allow complete adaptation between inoculated *Pseudomonas aeruginosa* and milk. Artificially contaminated milk was divided into seven groups (100 ml each) in the presence of a negative milk control inoculated with phosphate buffer saline (PBS) rather than *Pseudomonas aeruginosa*. Three artificially contaminated milk groups were inoculated with different concentrations of chitosan nanoparticles (50, 25, 15 mg/100ml). Another three artificial contaminated milk patches were inoculated with different concentrations of selenium nanoparticles (0.5, 0.3, 0.1 mg/100ml), and the last group served as positive control inoculated with *Pseudomonas aeruginosa* strain. Nanomaterials were mixed with the milk samples for 30 s. to ensure even mixing. All samples were transferred individually into sterile labeled test tubes and kept at 4 ± 1 °C for five days.

### Assessment of antibacterial activity of nanoparticles in the artificially contaminated milk

For the evaluation of antibacterial activity, a 100 µL aliquot of each milk treatment at different refrigerated storage times for five days treated with CsNPs and SeNPs at different concentrations in the presence of negative and positive milk control was diluted and plated in duplicate on *Pseudomonas* selective agar medium supplemented by glycerol and incubated at 37 °C for developing a greenish yellow colony. Finally, plates were examined, countable bacterial colonies (25–250 cfu) were recorded, and the results were reported as the average cfu/ml of three independent assays (Hernández–Díaz et al. 2021).

### Statistical analysis

The acquired data were subjected to a one-way ANOVA using SPSS (version 20; IBM, Chicago, IL, USA) to evaluate the differences between the tested treatments. When p ≤ 0.05, differences were deemed significant.

## Results

### Distribution of *Pseudomonas spp *and* P. aeruginosa *in milk and milk products

Preliminary survey for 100 random dairy sample, showing 45 positive sample for *Pseudomonas spp* were isolated. The highest mean average count was detected in raw milk samples 6.75 ± 1.12 log_10_cfu/ml, while the lowest *Pseudomonas spp* count was in ice cream samples with mean count 3.40 ± 0.79 log_10_cfu/ml as illustrated in Table 1.

**Table 1:** * Pseudomonas spp* count in different dairy products as raw milk, kareish cheese, yogurt and ice cream. *Pseudomonas spp* isolated on specific media showing greenish yellow colony

Log_10_ cfu/ml	Raw milk	Kareish cheese	Yogurt	Ice cream
Minimum count	4.54 ± 0.91	3.46 ± 0.94	3.43 ± 0.59	3.40 ± 0.79
Maximum count	6.75 ± 1.12	6.49 ± 1.31	4.76 ± 0.72	4.53 ± 0.78

The mean count expressed in log_10_cfu/ml

**Fig. 1:**
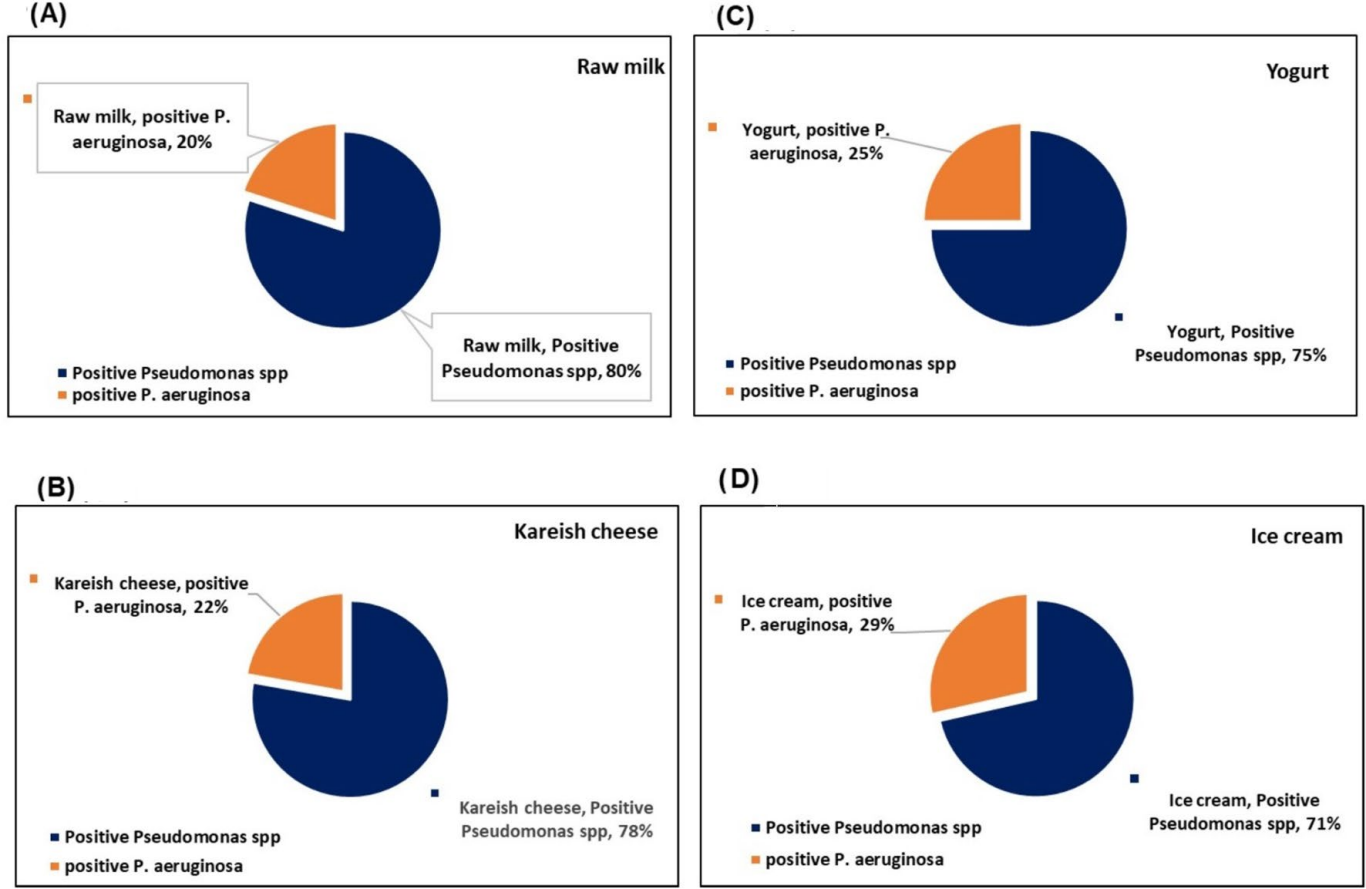
Prevalence of *P. aeruginosa* in a total 100 random sample incude raw milk, kareish cheese, yogurt and ice cream (25 sample each)

Figure 1 reported the prevalence incidence rate of *P. aeruginosa* in the positive detected isolates. The positive *Pseudomonas spp* in the total examined raw milk samples was 80% (20 positive sample from 25 examined milk sample), we found 20% are positive for *P. aeruginosa* as showed in Fig.1a. Kareish cheese is the second dairy product high in *Pseudomonas spp* incidence with 78% (14 positive sample from 25 examined kareish cheese sample), *P. aeruginosa* reperesnt 22% from the total positive samples as in Fig.1b. Comparing with the high prevalence of *Pseudomonas spp* in raw milk and kareish cheese, yogurt and ice cream showing the lowest prevealnce 75 and 71% respectively. Mean while, we observed that ice cream and yogurt showing the highest *P. aeruginosa* percent 29 and 25% from the positive sample as illustrated in Fig.1 c and d respectively.

### Morphology of nanoparticles

**Fig. 2:**
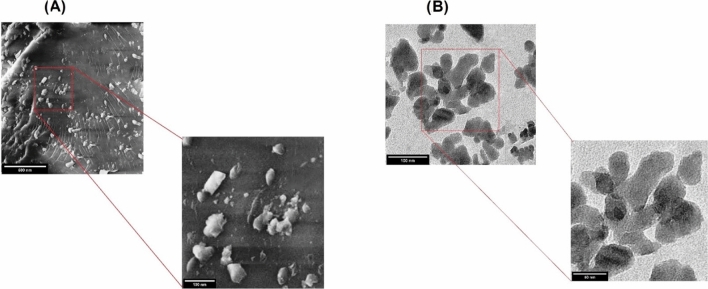
Showing the characteristics of chitosan nanoparticles (CsNPs) using electron microscope. **a** SEM image, **b** TEM image

Scanning and transmission electron microscopy are effective methods for determining nanostructure morphology and size (El-Naggar et al. 2020). The structures of both prepared nanoparticles were examined by SEM and TEM, as illustrated in Figs. 1 and 2.

The SEM image depict an investigation of the morphology of the synthesized CsNPs that showed rods’ smooth surface and platelet-like particles, as depicted in Fig.2a in accordance with (Pan et al. 2019).

**Fig. 3:**
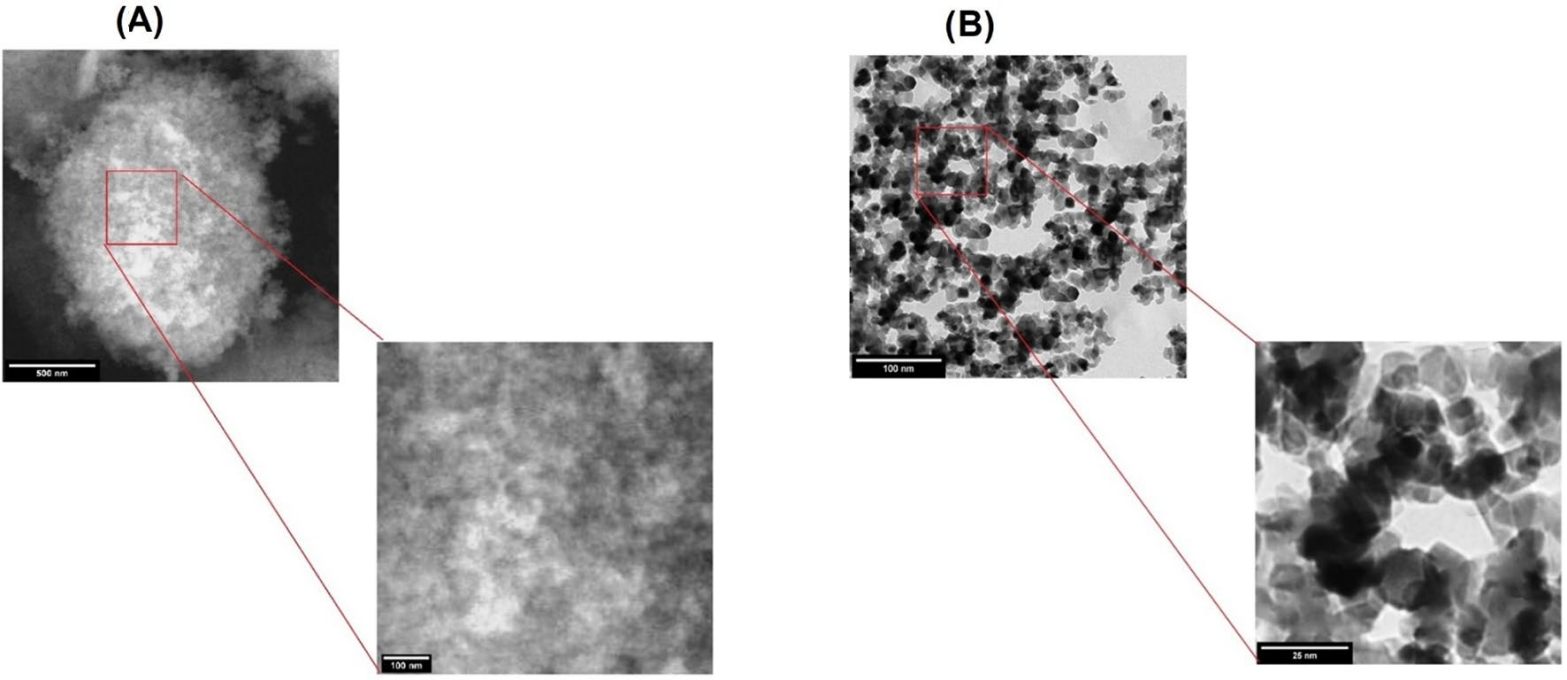
Showing the characteristics of selenium nanoparticles (SeNPs) using electron microscope. **a** SEM image, **b** TEM image

On the other hand, research using electron microscopy showed that SeNPs exhibited a good hexagonal shape with a smooth surface, as reported in Fig.3a. High surface energy and electrochemical characteristics can cause agglomeration (Peng et al. 2015). Fig.3b revealed that the TEM results also accurately determined the hexagonal structure of particles. Our findings concur with (Shar et al. 2019).

### Effect of prepared nanoparticles on * Pseudomonas aeruginosa* in milk

**Fig. 4:**
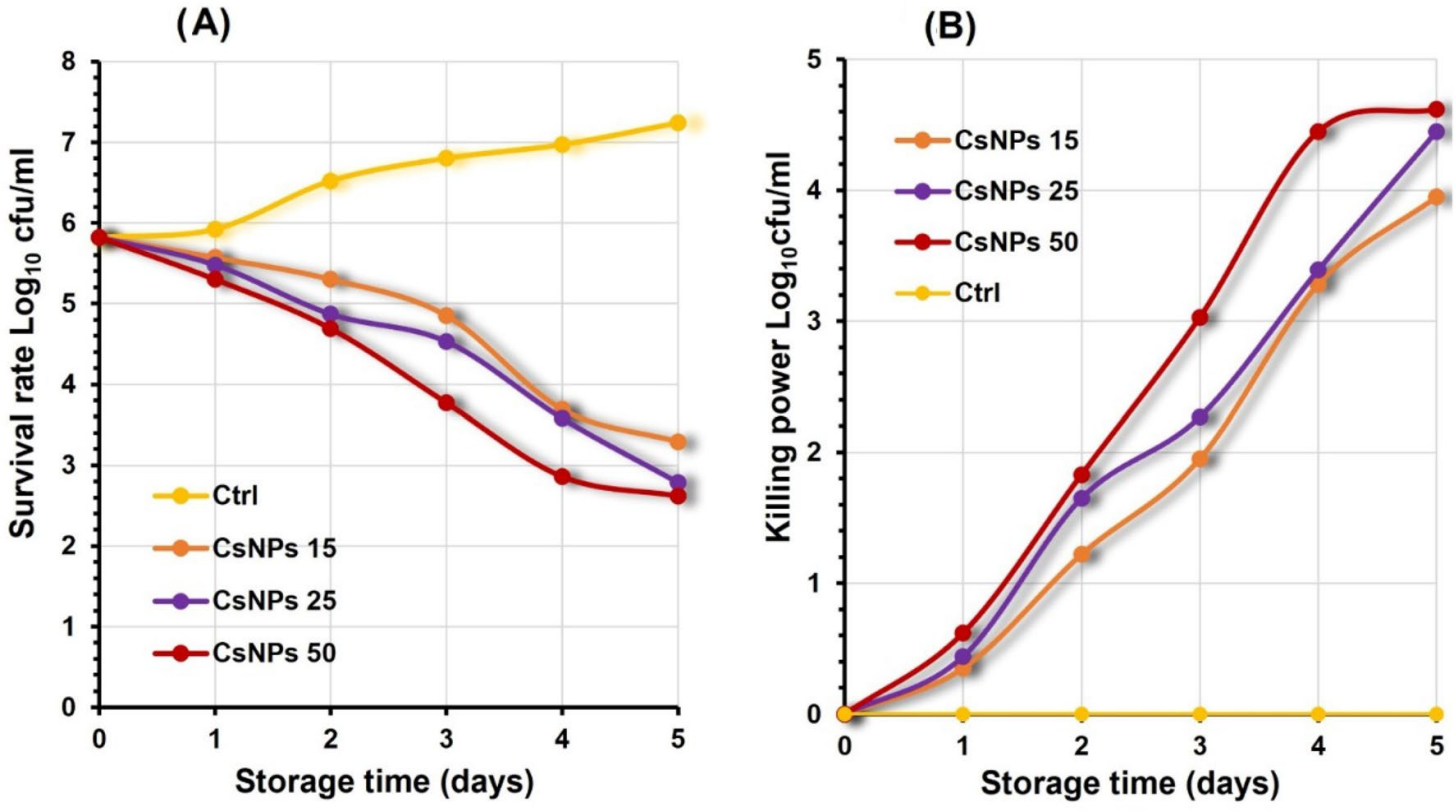
Illustrating Colony counting assay of *P. aeruginosa* in milk inoculated with different concentrations of chitosan nanoparticles (CsNPs) during five days cooling storage represented in Log_10_ cfu/ml. **a** growth survival rate of *P. aeruginosa* **b** killing power of *P. aeruginosa*. All values represent the mean ± standard deviation

**Fig. 5:**
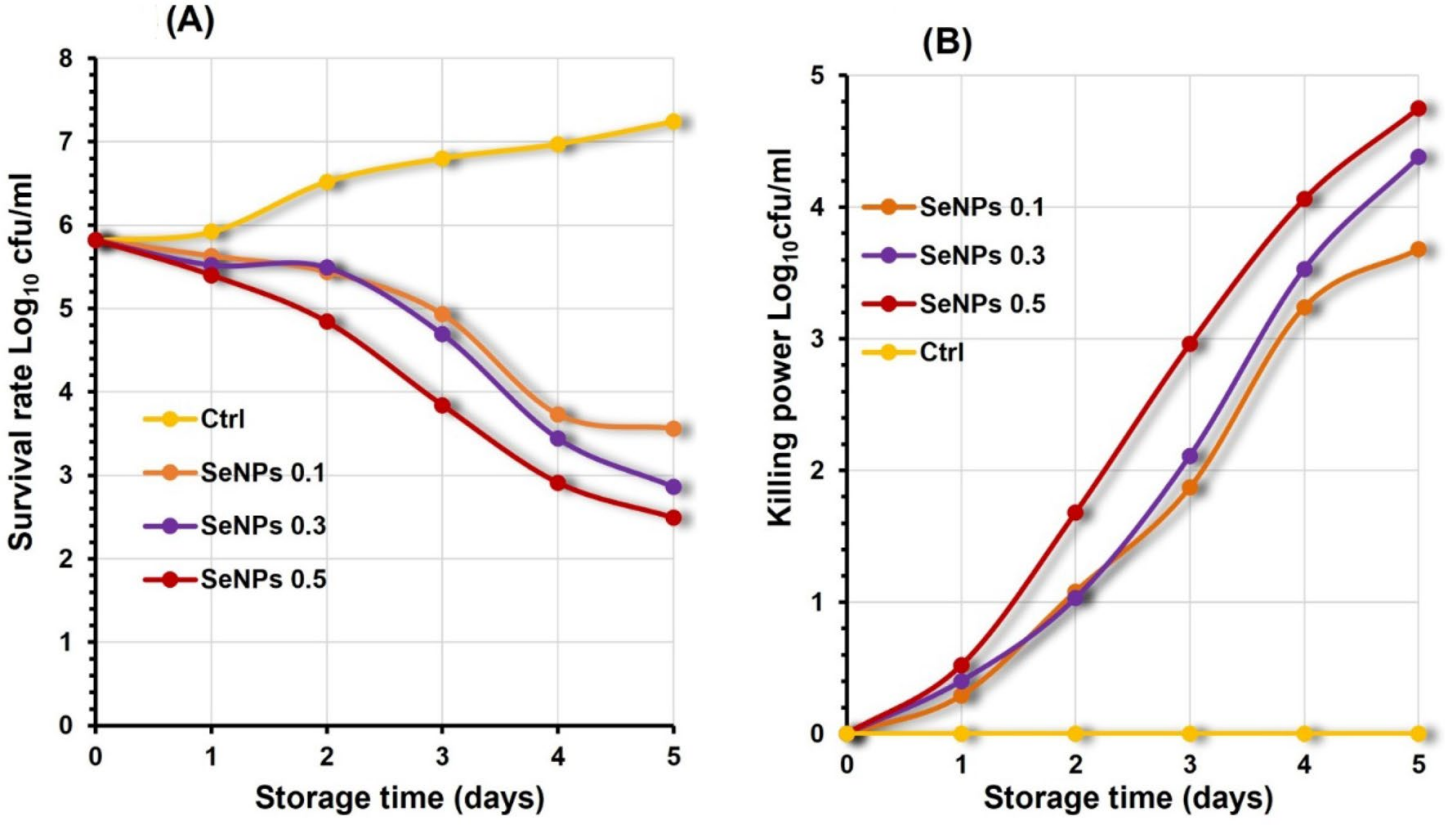
Illustrating Colony counting assay of *P. aeruginosa* in milk inoculated with different concentrations of selenium nanoparticles (SeNPs) during five days cooling storage represented in Log_10_ cfu/ml. **a** growth survival rate of *P. aeruginosa* **b** killing power of *P. aeruginosa*. All values represent the mean ± standard deviation

The major contributor to microbial contamination mainly causes food spoilage. Milk is the most perishable food and is easily susceptible to bacterial growth during various processing steps. An attempt was made to improve the safety by employing chitosan and selenium nanoparticles based on counting *P. aeruginosa* growth survival rate in milk. Generally, the *P. aeruginosa* control group demonstrated an increasing growth survival rate in buffalo milk during refrigerated storage, from a mean value of 5.82 ± 2.4 at zero day to 7.24 ± 2.57 log_10_cfu/ml on the fifth day from cooling storage. The result demonstrated that inoculated nanoparticles exhibit antibacterial properties against *P. aeruginosa* in a dose-dependent manner (Figs. 4 and 5).

Chitosan nanoparticles (CsNPs) at all concentrations effectively inhibit the growth survival rate of *P. aeruginosa*, as reported in Fig. 4a, with the best concentration of 50 mg/100 ml achieving a mean average growth survival rate of 2.62 ± 1.18 log_10_cfu/ml at the end of cold storage, followed by CsNPs 25 and CsNPs15 with growth survival rates 2.79 ± 0.76 and 3.29 ± 1.76 log_10_cfu/ml respectively. In contrast, the control bacterial growth rate reaches 7.24 ± 2.57 log_10_cfu/ml. Similarly, we discovered that CsNPs at concentration 50 mg/100ml exhibits the highest killing power with a mean value of 4.62 ± 1.37 log_10_cfu/ml on the fifth day at cooling storage compared with zero killing power at the control group, as shown in Fig. 4b.

At all concentrations, selenium nanoparticles (SeNPs) exhibited potent antibacterial activity against *P. aeruginosa*, as illustrated in Fig. 5a, with SeNPs at concentration 0.5 mg/100 ml being the best concentration to effectively inhibit *P. aeruginosa* growth survival rate with a mean average 2.49 ± 11 log_10_cfu/ml at the end of refrigerated storage, followed by SeNPs 0.3 and SeNPs 0.1 with mean growth survival rate 2.86 ± 0.76 and 3.56 ± 2.32 log_10_cfu/ml respectively. On the other hand, the control group showing bacterial growth reached 7.24 ± 2.57 log_10_cfu/ml. As reported in Fig. 5b, we noticed that SeNPs at concentration 0.5 mg/100 ml had the highest killing power with a mean value of 4.75 ± 1.56 log_10_cfu/ml on the fifth day from cold storage as opposed to the control group, which had no killing power.

**Fig. 6:**
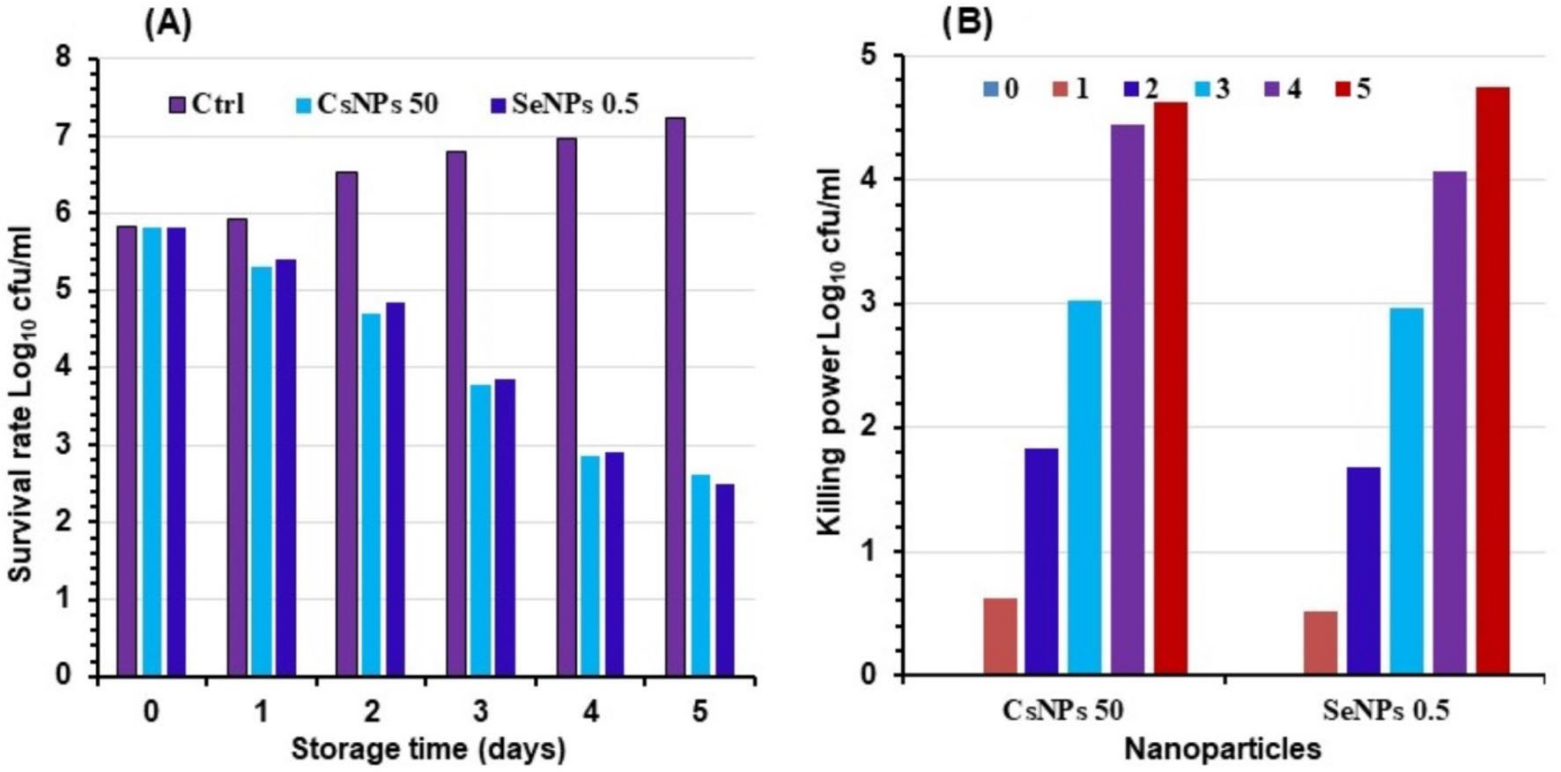
Illustrating Colony counting assay of *P. aeruginosa* in milk inoculated with specific concentrations of chitosan nanoparticles (CsNPs 50 mg/100ml) and selenium nanoparticles (0.5 mg/100ml) during five days cooling storage represented in Log_10_ cfu/ml. **a** growth survival rate of *P. aeruginosa* **b** killing power of *P. aeruginosa0*

In buffalo milk inoculated with specific concentrations of CsNPs (50 mg/100ml) and SeNPs (0.5 mg/100ml), we compared the development growth patterns of *P. aeruginosa*. The growth survival rate of *P. aeruginosa* throughout cooling storage days, as shown in Fig. 6a, and the effectiveness of the killing power for both concentrations, as shown in Fig. 6b, did not differ significantly from one another.

## Discussion

Regarding being a great source of nutrients, including high-quality bioavailable protein, lactose, milk fat and calcium, as well as a variety of bioactive peptides, immunological factors, growth factors, enzymes, and hormones, milk is recognized as nature’s most nearly perfect food (Li et al. 2018). These favorable nutrients encourage bacterial development and reproduction, quickly resulting in milk spoilage, reducing the milk’s organoleptic quality, shortening its shelf life, and increasing the risk of foodborne infections (Dash et al. 2022).

Furthermore, because *Pseudomonas spp.* and other psychotropic organisms are growing, contamination may occur when raw milk is delivered in refrigerated tanks at 4–7 °C (Fernandes 2009). Naturally derived products and ingredients have been employed as unique antimicrobial strategies for controlling pathogenic bacteria and spoilage and improving food quality (Kang et al. 2020).

The current study is the second part of surveillance investigation for detection the virulence the genes of *P. aeruginosa* according to (Attia et al. 2022). As regarded in the findings, raw milk and kareish cheese were contaminated with highest count *Pseudomonas spp* while ice cream and yogurt showed the lowest *Pseudomonas spp* count. Although the low prevalence count of *Pseudomonas spp* in ice cream and yogurt, the products contaminated with potent virulent *P. aeruginosa.* These results could be attreibuted to the storage conditions (time, temperature and place) are the most contributor in the high *Pseudomonas spp* count in raw milk and kareish cheese (De Jonghe et al. 2011; Narvhus et al. 2021). Our results were in agreement with De Jonghe et al. (2011).

Although milk subjected to thermal treatment before manufacture yogurt and ice cream, they were also contaminated by *Pseudomonas spp* which might be due to post-pasteurization contamination at packaging or during the domoinat psychrotrophic bacteria in storage refrigerator temperature (Eneroth et al. 1998).

It is worthy noted that, *Pseudomonas aeruginosa* is one of common Gram-negative bacterial pathogen that cause many health risk infections with identified antimicrobial resistance (Reynolds and Kollef 2021). Our study reveals high prevalence *P. aeruginosa* contamination in yogurt and ice cream, that is why the search for natural bioprservatives (chitosan and selenium nanoparticle) is very critical issue.

As reported in our current investigation, we use three different modest concentrations of CsNPs (50, 25, and 15 mg/100ml) and SeNPs (0.5, 0.3 and 0.1 mg/100ml), these small concentrations are unaffected the color, texture and overall appearance of milk samples. The raw buffalo milk’s white colour can obscure the characteristic coloration of the selenium nanoparticles concentrations used (data not shown). Therefore, the addition of the nanoparticles utilized in our current investigation does not pose a risk to the raw milk’s physical characteristics.

We found chitosan nanoparticles acquired potent antibacterial activities. This may be attributed to chitosan acquiring a positive charge that can interact with bacteria’s negative charge in their membranes. This electrostatic interaction results in cell membrane lysis, which alters bacterial cell permeability (Rabea et al. 2003; Tripathi et al. 2008). This is a plausible explanation for the antibacterial activity of chitosan nanoparticles besides its nano diameter of particles, according to (Tsai and Su 1999), who reported that the antibacterial mechanism requires a reduction in the size of chitosan to allow penetration into the cellular system.

Chitosan nanoparticles were synthesized using a variety of techniques, including ionic gelation (Calvo et al. 1997), where the electrostatic interaction between the negatively charged group of tripolyphosphate (TPP) and the positively charged group of chitosan was performed by the ion gelation process. The size and surface charge of nanoparticles could be altered by varying the chitosan to TPP ratio. Since the amine moieties of chitosan may cross-link with the phosphine residues of TPP during the reaction, forming nanoparticles. The chitosan molecular structure is modified to enhance its physicochemical properties at the nanoscale, including increased water solubility, antibacterial activity, and other sensory properties (Geng et al. 2005).

On the other hand, selenium nanoparticles have developed several distinctive physicochemical characteristics that make them appealing for technological uses in many fields of biomedicine and photoelectrochemistry (Peng et al. 2015; Geoffrion et al. 2020). It has acquired many advantages, including minimal toxicity, good degradability, and exceptional anticancer, antiviral, and antimicrobial properties (Wadhwani et al. 2016; Hosnedlova et al. 2018).

Our findings cleared that selenium nanoparticles have good antibacterial action, which may be attributed to the increased release of selenium ions to damage the bacterial structure. Our findings could be supported by a study supported by ( Lin et al. 2019), who supported our concept and found that selenium nanoparticles exhibited potent activity against multidrug-resistant bacterial infections and might be employed as a promising prospect as an antibiotic alternative. Zhang et al. (2021) demonstrated the antibacterial activity of selenium nanoparticles showed potent antibacterial against Gram-negative bacteria than Gram-positive bacteria after 12 h. Cell leakage studies revealed that there were polysaccharides and proteins released from the cells after reacting with selenium nanoparticles, which is thought to be the cause of the powerful killing capability. It was discovered that the rupture of cell walls and alterations in the permeability of the membrane were to blame for the leakages of proteins and polysaccharides. Furthermore, the intensity of free radicles changed, suggesting that oxidative damage may be a major factor in antibacterial activities.

Recent studies reported weaned piglets fed diets enriched with the nanoparticles of chitosan for a period of 28 days showed enhancements in gut microbiota and immunological responses. The findings demonstrated that dietary supplementation with chitosan nanoparticles altered the composition of colonic microflora, increasing the level of some likely beneficial intestinal bacteria while restricting the growth of prospective bacterial pathogens. These findings indicate that fortification with CsNPS enhanced immune function, reduced immunological stress, and controlled intestinal ecology in weaned pigs by reducing inflammatory intestinal damage. Urging its use as a functional feed supplement (Xu et al. 2020, 2023). Additionally, SeNPs has been recommended as a healthier and more effective vehicle for delivering selenium for physiological demands owing to its unique biological features. Diet Supplemented with SeNPs may drastically change the gut microbiota’s behavior (Qiao et al. 2022). It was reported that incorporating nanoparticles as a preservative food additive with the goal of boosting food attributes and expiration date has dramatically increased recently. In accordance with a new investigation, SeNPs drastically decreased the prevalence of caecal pathogens without having a substantial negative impact on the overall microbial population. It also had a moderate effect on the diversity forms and compositional component of the poultry caecal microbiota (Gangadoo et al. 2019). Another study findings discovered the potential applications, substantial bioavailability and low toxicological profile of selenium nanoparticles and its utilization in lowering the level of oxidative stress in tissue of animals, these particles receiving growing popularity, the most recent study involves adding SeNPs as a food supplement to fish diets in varied doses. Since the intestinal microbial community’s total diversity was low, using SeNPs as a food additive might help aquatic animals’ nutritional health (Jia et al. 2022). However, according to previous studies and the utilization of low concentrations of selenium and chitosan nanoparticles in our current, might suggest the neglectable modifications in human gut microbiota. We recommend more future research to determine the impact of utilized nanoparticles in milk on consumer gut microbiota.

These studies strongly imply that chitosan and selenium nanoparticles can serve as functional ingredients in food bio-preservation against *P. aeruginosa* besides, maintaining the good hygienic measures under which milk is produced to decrease the risk of against *Pseudomonas spp* contamination during packaging or storage.

## Conclusion

*Pseudomonas aeruginosa* is opportunistic Gram negative bacteria, It considers a major cause for most chronic diseases with antimicrobial resistance. *P. aeruginosa* contamination may have contributed to milk spoilage and different related milk products due to post – pasteurization contamination or bad storage. Our current investigation elucidated the antibacterial activity of synthesized chitosan and selenium nanoparticles (CsNPs and SeNPs) at various concentrations on the growth patterns of *P. aeruginosa* in milk during refrigerated storage. The available findings revealed no discernible difference in the killing power between CsNPs (50 mg/100ml) and SeNPs (0.5 mg/100ml), where the mean killing activity values were 4.62 ± 1.37 and 4.75 ± 1.56 log_10_cfu/ml respectively, at the fifth day of cooling storage. As described by SEM and TEM, ion gelation and green synthesis effectively convert chitosan and selenium into nanoparticles that could be applied in milk, respectively. Our study targets the efficacy of nanoparticles on *P. aeruginosa* as an antibacterial activity. So, we can recommend studying the fate of these nanoparticles with further nanoparticles characterizations in milk as a future study. Therefore, milk containing chitosan and selenium nanoparticles may be a promising candidate for natural antibacterial agents to improve milk safety in a condition, maintaining the hygienic measures for milk production and storage.

## Data Availability

The data presented in this study are available on request from the corresponding authors.
